# Patient-Derived Tumor Organoids: New Progress and Opportunities to Facilitate Precision Cancer Immunotherapy

**DOI:** 10.3389/fonc.2022.872531

**Published:** 2022-04-05

**Authors:** Ji Wang, Chao Chen, Lu Wang, Mingjun Xie, Xinyang Ge, Sufan Wu, Yong He, Xiaozhou Mou, Chenyang Ye, Yi Sun

**Affiliations:** ^1^Center for Plastic & Reconstructive Surgery, Department of Plastic & Reconstructive Surgery, Zhejiang Provincial People’s Hospital (Affiliated People's Hospital, Hangzhou Medical College), Hangzhou, China; ^2^Department of Colorectal Surgery and Oncology, Key Laboratory of Cancer Prevention and Intervention, Ministry of Education, The Second Affiliated Hospital, Zhejiang University School of Medicine, Hangzhou, China; ^3^Cancer Institute (Key Laboratory of Cancer Prevention and Intervention, China National Ministry of Education), The Second Affiliated Hospital, School of Medicine, Zhejiang University, Hangzhou, China; ^4^Cancer Center, Zhejiang University, Hangzhou, China; ^5^State Key Laboratory of Fluid Power and Mechatronic Systems, School of Mechanical Engineering, Zhejiang University, Hangzhou, China; ^6^Key Laboratory of Materials Processing and Mold, Zhengzhou University, Zhengzhou, China; ^7^College of Letters and Science, University of California, Los Angeles, Los Angeles, CA, United States; ^8^Key Laboratory of 3D Printing Process and Equipment of Zhejiang Province, College of Mechanical Engineering, Zhejiang University, Hangzhou, China; ^9^Department of Medical Oncology, The Second Affiliated Hospital of Zhejiang University School of Medicine, Hangzhou, China

**Keywords:** tumor organoid, tumor microenvironment (TME), precision medicine, multi-omics, exosome (vesicle), CRISPR, proteomics

## Abstract

Cancer immunotherapy has revolutionized the field of cancer treatment in recent years. However, not all patients receiving cancer immunotherapy exhibit durable responses, and reliable, high-throughput testing platforms are urgently needed to guide personalized cancer immunotherapy. The ability of patient-derived tumor organoids to recapitulate pivotal features of original cancer tissues makes them useful as a preclinical model for cancer research and precision medicine. Nevertheless, many challenges exist in the translation of tumor organoid research to clinical decision making. Herein we discuss the applications of patient-derived tumor organoid models and the advances and potential of using complex immune-organoid systems as testing platforms to facilitate precision cancer immunotherapy. In addition, we highlight intriguing applications of tumor organoids with novel multi-omics in preclinical cancer research, highlighting genetic editing, proteomics, and liquid biopsy.

## Introduction

Despite developments in early detection and treatment in the past decade, cancer remains the second leading cause of death worldwide (1). Recently, the emergence of cancer immunotherapy has revolutionized conventional cancer therapeutics and rejuvenated the field of cancer immunology (2–5). Nevertheless, only a select group of cancer patients have achieved marked clinical responses to cancer immunotherapy (6–9). The pressing need to improve cancer immunotherapies has brought great attention to the tumor immune microenvironment (TIME), whose study requires robust and faithful preclinical research models recapitulating patient-specific tumor-immune interactions.

The TIME, including immune cells and cancer-associated fibroblasts (CAFs), greatly fosters carcinogenesis and tumor progression and influences the therapeutic responses of malignant cells (10–12). However, it was previously difficult to model this TIME in experiments (13). Cancer cell lines and patient-derived xenograft models (PDXs) suffer from several limitations. The former fail to adequately reflect the heterogeneity of tumor epithelial cells (13), and the latter are based on the murine immune system, which cannot replicate the tumor-immune interactions in humans (14). Patient-derived tumor organoids (PDTOs) have emerged as a useful model that can maintain tumor epithelial cells in a near-native state (15). PDTOs are able to maintain the heterogeneity of original cancers and can recapitulate the human TIME (16–18), thus providing an intriguing opportunity to facilitate precision cancer immunotherapy.

In this paper, we will discuss the state of the art of organoids in cancer immunological research. The limitations and prospects of this complex tumor organoid culture system are presented. We propose the potential applications of complex tumor organoids as testing platforms for various cancer immunotherapeutic approaches including antibody-based immunotherapy, oncolytic virus therapy and adoptive cell transfer therapy. We also highlight the intriguing combination of PDTOs with cutting-edge multi-omics and their applications in investigating cancer immunobiology and developing immunotherapy drugs.

## Patient-Derived Tumor Organoids

Organoids are 3D self-organized structures derived from adult tissue stem cells, embryonic stem cells, or induced pluripotent stem cells that mimic key structural and functional features of their *in vivo* counterpart organs (18–20). In 2009, Hans Clevers’s group developed the first organoids from mouse intestinal stem cells (21), which established the starting point for other culture protocols for mouse and human tissue-derived organoids. The development of tumor organoid culture has allowed the application of PDTOs to test and predict drug responses in the context of precision cancer treatment. Currently, tumor organoid biobanks have been established from various types of cancer, including breast (17), lung (22, 23), colorectum (24–28), stomach (29–31), liver (32, 33), pancreas (34), ovary (35, 36), prostate (37), and brain (38). Although these epithelial-only PDTOs are generally available, their lack of immune and other nonimmune components of the TIME impedes immunotherapy assessment, such as checkpoint inhibition blockade and adaptive T-cell therapy. Therefore, significant effort is needed to optimize the tumor organoid culture system, forging a path toward organoid-guided personalized cancer immunotherapy.

## Recent Advances in Complex Tumor Organoid Culture

An increasing number of studies have focused on the essential factors of organoid development and novel organoid culture methods to recapitulate the TIME, facilitating the basic research and clinical translation of immuno-oncology. In this section, we describe the recent advances in complex tumor organoid culture systems (**Table 1**).

**Table 1:** Overview of currently established tumor organoid-immune cell co-culture systems.

Co-culture approach	Tissue of origin	Sample type	Species	Immune cell type	Duration (days)	Functionality	Refs
Holistic approach	MC38 CRC cell line/Melanoma	Cell implantation orthotopically/Surgical specimens	MDOTS/PDOTS	T cells, B cells, granulocytic, monocytic lineages, dendritic cells,	9 days	Preserve immune cell reaction to immune checkpoint inhibitors	(39)
	MC38 CRC cell line	Subcutaneous mouse tumors	MDOTS	T cells	5 days	Preserve immune cell reaction to CDK4 and CDK6 inhibitors plus immune checkpoint inhibitors	(40)
	Colon, pancreas, and lung (14 distinct tissue sites)	Subcutaneous mouse tumors/Surgical specimens	MDOTS/PDOTS	Macrophages, T cells, NK cells, and B cells	30 days	Preserve the TCR repertoire of the original fresh tumor	(41)
	CRC or lung cancer	Surgical specimens	PDOTS	CD45^+^ tumour-resident leukocytes	>10 days	*In vitro* survival of CD45^+^ cells	(42)
	Breast	Surgical specimens	PDOTS	Peripheral blood and tumour-derived γδ T cells	2-3 w	Preserve γδ T cell activation and tumour cell line cytolysis	(43)
Reconstitution approach	Gastric cancer	Triple-transgenic mouse model	MDOTS	CD8+ splenocytes and bone marrow-derived DCs	2 days	Organoid cytolysis	(44)
	Pancreatic cancer	Surgical specimens	PDOTS	peripheral blood lymphocytes & CAFs	6 days	Tumor-dependent lymphocyte infiltration and activation of myofibroblast-like CAFs	(45)

MDOTS, murine-derived organotypic tumor spheroids; PDOTS, patient-derived organotypic tumor spheroids;

CAFs, cancer-associated fibroblasts.

### Adding Immune Cells to Organoid Culture

Currently, there are two conceptually different approaches to organoid–immune cell coculture models: the reconstitution approach expands tumor organoids and immune cells separately and then generates a coculture system with both components, whereas the holistic approach uses tumor organoids cultured directly from tumors while retaining endogenous immune cells (16, 46) (**Table 1**).

Reconstitution approaches initially expand organoids and immune cells separately and then establish cocultures for the investigation of organoid-immune cell interactions. The reconstitution of tumor organoids with various immune cell populations has been explored (**Table 1**). One study reported a triple coculture of mouse gastric tumor organoids with dendritic cells (DCs) and cytotoxic T lymphocytes (CTLs) (44). The stimulated CTLs resulted in significant death of gastric tumor organoids in the presence of an anti-PD-L1 neutralizing antibody (44). Additionally, PDTO-T cell cocultures hold the potential to predict the functionality of tumor-infiltrating lymphocytes (TILs) after immune checkpoint blockade. In a proof-of-principle study, the authors cocultured human colorectal cancer organoids with TILs using a reconstitution approach (47). The authors exposed the coculture to anti–PD-1 antibody and identified partial restoration of antitumor immunity in TILs with increased PD1 expression (47), revealing that these coculture assays have potential as a platform to evaluate the efficacy of cancer immunotherapy.

In contrast to reconstitution approaches, which add exogenous immune cells into epithelial-only tumor organoids, holistic approaches expand and activate endogenous immune cells within tumor organoids as a cohesive unit. A study in 2016 reported that intraepithelial lymphocytes were retained within organoids derived from human epithelial breast tissue (43). The authors then treated organoids for up to 4 weeks with aminobisphosphonate drugs that have been proven to selectively activate Vδ2^+^ T cells, a subset of IFNγ-producing T cells. In a subsequent experiment, stimulated Vδ2+ T cells from breast organoids produced the antitumor cytokine IFNγ and efficiently killed breast carcinoma cells (43). In early 2018, two groups described 3D microfluidic-based culture to recapitulate anti-PD1/PDL1 cancer immunotherapy using mouse-derived and patient-derived organotypic tumor spheroids (MDOTS and PDOTS, respectively) (39, 40). Tumor spheroids together with endogenous lymphocyte and myeloid populations could be preserved for short-term (5-9 days) culture, allowing the investigation of endogenous immune–tumor interactions (39, 40). Later in the same year, one study described a sophisticated air-liquid interface (ALI) organoid culture method that enabled the coculture of the original tumor epithelium with its diverse endogenous immune cells (41). The authors showed that a diversity of endogenous immune cell types, including tumor-associated macrophages, T cells [T helper (Th), cytotoxic (Tc), regulatory (Treg), and exhausted (Tex)], natural killer (NK) cells, and B cells, were successfully cultured for up to 30 days in ALI organoid cultures (41). Strikingly, the ALI PDTOs could preserve the T cell receptor (TCR) heterogeneity found in the original tumor and model immune checkpoint blockade, which led to the proliferation and activation of tumor antigen-specific T cells and subsequent tumor cytotoxicity (41).

### Adding Cancer-Associated Fibroblasts

CAFs account for a large proportion of the tumor stroma and play considerable roles in the TIME (48–50). Therefore, it is important to include CAFs in the culture system of tumor organoids. Indeed, PDTOs and CAFs have recently been employed in 3D coculture systems to investigate the reciprocal interaction between tumor cells and CAFs (51–53). CAFs have been utilized to supplement PDTO cultures using a reconstitution approach (52–54). One study explored CAF heterogeneity by coculturing pancreatic cancer organoids and CAFs and identified two spatially separated subtypes of CAFs with distinct protein expression profiles: high α-smooth muscle actin (αSMA)-expressing myofibroblast-like CAFs proximal to tumor cells and high IL-6-expressing inflammatory CAFs distally located from neoplastic cells (53). The dynamic tumor-stroma interaction was investigated in a 3D coculture system of lung squamous carcinoma (LUSC) organoids with CAFs and extracellular matrix (ECM) (55). Intriguingly, the authors showed that CAFs could override cell intrinsic oncogenic changes in determining the disease phenotype in the LUSC setting (55). The ability to retain the heterogeneity and phenotype of the original tumor tissue makes the 3D coculture system of PDTOs and CAFs a promising model for tumor immune microenvironment research.

### Adding Vasculature

Another major issue in the current organoid system is the lack of vascular circulation. Without a blood supply, organoids can grow only to a limited size, beyond which the center of the organoid would develop necrosis (56, 57). To overcome this challenge, developments in organoid vascularization and perfusion are required to maintain the complexity and scale of organoids.

Organoid vascularization could be generated by the transplantation of organoids into vasculature-rich animal tissue, including chicken chorion allantois membrane models, with the host vasculature integrating into the organoids (58, 59). The methods of adding vasculature *in vitro* include the layer-by-layer deposition of endothelial cells and the selective removal of material to form tubular voids that are connected to perfusion networks (60). Moreover, vasculature is also induced in organoid-endothelial cell cocultures in microfluidic devices (60). In one recent study, 3D tumor spheroids were integrated with human umbilical vein endothelial cells (HUVECs) and normal human lung fibroblasts (nhLFs) in a fibrin gel, which developed a perfusable vasculature *in vitro* (61).

### Adding Extracellular Matrix

The *in vivo* ECM is a dynamic polymer network that not only provides structural support but also delivers biochemical signaling cues (62, 63). In relation to cancer research, the ECM plays critical roles in tumor growth, invasion, metastasis, and metabolism (64, 65). Therefore, it is crucial to integrate an appropriate ECM into tumor organoid culture models. Over the last decade, the most commonly used matrix for the culture of tumor organoids has been basement membrane extracts (BMEs) (66). The BMEs have been commercially available under the trade name Corning Matrigel, a solubilized basement membrane matrix secreted by Engelbreth-Holm-Swarm (EHS) mouse sarcoma cells. BMEs that include ECM proteins (laminin, collagen IV, and entactin) are simple to prepare and use in organoid culture. Although BMEs have provided a tumor-relevant environment for human tumor organoid culture, several limitations hinder our understanding of organoid-ECM interactions, including extensive batch-to-batch variability, xenogenic contamination, ill-defined ECM components, and poor control of mechanical properties (67). Collagen is the most abundant structural ECM component in human tumor tissues. Collagen type I matrices are also widely used as scaffolds for tumor organoid studies because they share biochemical and biophysical features of the TIME, such as cell adhesion sites and stiffness (68). Tumor organoids with a collagen type I matrix could be utilized for the investigation of invasive cell phenotypes (69, 70). Nevertheless, as collagen is often animal derived, collagen type I matrices suffer from similar limitations to BMEs. Thus, it is imperative to develop new ECM materials to replace the current animal-derived matrices. Engineered matrices are promising alternatives for scaffolds in tumor organoid models, offering well-defined, tunable ECMs with high batch-to-batch reproducibility (71). Nevertheless, engineered matrices also have some limitations, including low culture efficiency and a lack of sufficient spatiotemporal control to model the dynamics of the TIME. Overall, further efforts are still required to develop the optimal ECM for tumor organoid culture, facilitating a more complete understanding of cancer-ECM interactions *in vitro*.

## Complex Organoids and Immunotherapeutics

An ideal preclinical platform to test cancer immunotherapies requires a cancer–immune cell coculture which reflects the cellular distribution of the original tumor and recapitulates the response to immunotherapeutic. Recent advances in complex tumor organoids have shown this organoid system could be used as efficient and pivotal platforms to assess the efficacy of cancer immunotherapy and the identification of novel combination treatment strategies.

### Antibody-Based Immunotherapy

Antibody-based immunotherapy is a major form of cancer immunotherapeutics that can specifically limit cancer cell survival and activate the immune system to eradicate cancer cells (72). Currently, tumor organoids have mainly been employed as preclinical models to investigate the efficacy of antibody-based checkpoint blockade immunotherapy. Using either reconstitution or holistic approach, the efficacy of PD-1/PD-L1 immunotherapy could be recapitulated in complex organoid culture system in the presence of functional immune cell populations (41, 45). Intriguingly, the immune–tumor organoids system could also be used to identify novel strategies for antibody-based combination cancer treatments. Small molecule inhibitors such as TBK1/IKKe inhibitor or CDK4/6 inhibitor were reported to synergize with PD-1 blockade and lead to enhanced tumor killing (39, 40). Bispecific immunomodulatory antibodies could simultaneously bind two different antigens located on cytotoxic cell and target tumor cell respectively, resulting in tumor cytotoxicity. Cibisatamab is a bispecific antibody designed to target CD3 on T cells and CEA in colorectal cancer cells. In one recent study, the complex cancer organoids were used to identify potential novel strategy for enhanced therapeutic effect of cibisatamab (73).

### Oncolytic Virus Therapy

Oncolytic viruses that preferentially infect and replicate in cancer cells, provide an intriguing immunotherapeutic option for cancer patients (74). Oncolytic viruses can not only result in direct destruction of cancer cells, but also trigger host anti-tumor immune system responses (75). Several groups used tumor organoids to evaluate the efficacy of oncolytic virus therapy in preclinical settings. These studies demonstrated that oncolytic adenovirus could show selective replication in PDTOs while not in organoids derived from normal tissue, and tumor organoids are ideal preclinical models to predict responses to oncolytic adenovirus therapy (76–78). However, these studies failed to investigate oncolytic virus therapy in complex immune–organoids. One recent study first described the efficacy of a novel oncolytic adenovirus treatment in PDTOs with various immune cell populations. In order to activate multiple immune effector populations including neutrophils and natural killer cells, the authors engineered a Fc-fusion peptide against PD-L1 consisting of a cross-hybrid Fc region containing constant regions of an IgG1 and an IgA1. This Fc-fusion peptide was cloned into an oncolytic adenovirus, and enhanced oncolytic efficacy was observed in complex immune–organoids platform (79).

### Adoptive Cell Transfer Therapy

Adoptive cell transfer therapy represents an important alternative to immune checkpoint inhibitors and uses genetically engineered T cells with chimeric antigen receptors (CARs) or high-affinity T cell receptors (TCRs) recognizing tumor-associated antigens (80). In this scenario, antitumor lymphocytes are expanded ex vivo and then given back to the patients. While CAR-T cells targeting CD19 show prominent effects in hematological malignancies including B cell lymphoma and acute lymphoblastic leukemia, efficacy in solid tumor remains elusive. Complex organoids have shown great potential to serve as efficient platforms for evaluation of CAR cell efficacy. PDTOs have now been used to test specific tumor killing of CAR-NK92 targeting EGFRvIII or FRIZZLED in colorectal cancer setting (81). Additionally, PDTOs could be utilized as culture platforms to enrich tumor reactive T cells and induce more effective anticancer immune responses (82).

## Combination of PDTOs With Multiomics

**Figure 1:**
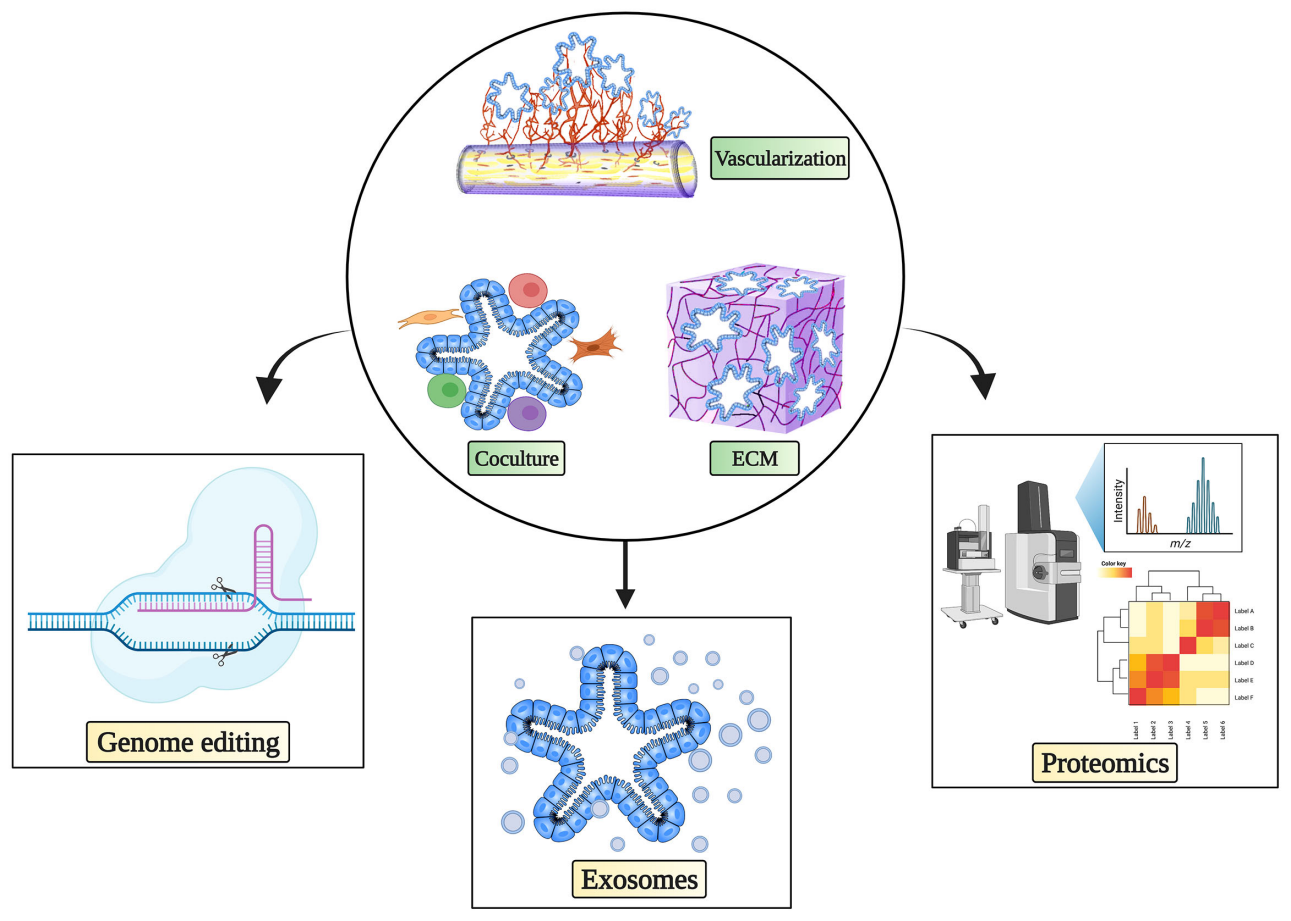
Complex culture system and possibilities for tumor organoids in cancer immunotherapy research. Complex immune organoid culture systems including fibroblasts, various immune cells, and vasculature in addition to tumor organoids could be leveraged to serve as platforms for testing cancer immunotherapy. Various state-of-the-art technologies can be used in combination with complex tumor organoid culture systems to propel precision medicine. The figure was generated on Biorender.com.

Recent advances in organoids have not only facilitated biobank-based disease modeling, cancer therapeutic strategies, and personalized medicine (15) but also revolutionized the field of cancer studies by improving the understanding of mechanisms and disease modeling at the molecular level (18, 83). Nevertheless, based on current knowledge and applications, further improvements on the road to tumor organoid-based clinical decision making are still required. In this section, we will discuss the latest applications and promising prospects of tumor organoids in various omics disciplines that could help move precision medicine forward (**Figure 1**).

### Genetic Engineering

Genetic engineering, especially the CRISPR–Cas9 system, has substantially improved genetic modification and screening in both *in vitro* and *in vivo* human cancer models, such as cell lines and mouse models, revealing previously unknown cancer drivers (84–90). Nevertheless, it is difficult for these preclinical models to accurately recapitulate *in vivo* tumor biology. To this end, there has been a growing interest in performing CRISPR–Cas9 genome editing in tumor organoids. In 2013, Hans Clevers’s group was the first to implement CRISPR–Cas9 technology in an organoid model (91). They successfully corrected the CFTR gene in intestinal organoids, demonstrating that the CRISPR/Cas9 system is feasible and efficient for genome editing in patient-derived organoids (91). In one study, Han and coworkers performed genome-wide CRISPR screens in both 2D and 3D lung cancer models and found that screening in 3D models captured the characteristics of oncogenes and tumor suppressor genes more accurately than the use of 2D models (92). Interestingly, the authors reported that the knockout of the cancer driver gene CREBBP exerts a positive growth impact on a 3D model but a negative growth effect in a 2D cancer cell line (92). CRISPR–Cas9 could also be used to investigate the clonal evolution of carcinogenesis. Successful multihit oncogenic transformation of normal-tissue-derived organoids to carcinoma has been achieved by introducing simultaneous or sequential oncogenic mutations into tissues such as breast, stomach, pancreas and colon (31, 93–96). Although several limitations need to be addressed including heterogeneous growth rates of organoids and single-guide RNA coverage (97), cancer organoids could serve as a promising platform for CRISPR–Cas9-mediated genome editing and large-scale screens to improve the efficacy of cancer immunotherapy.

### Proteomics and Immunopeptidomics

Mass spectrometry (MS)-based proteomics shows great promise to yield important insights for cancer therapy, yet poor resolution, the need for large amounts of samples, and the absence of high-throughput capacity are limiting factors. Recent advances in sample processing, separations and MS instrumentation highlight the possibility of personalized proteomics (98). The potentially unlimited supply of well-characterized patient material makes organoids a unique platform for personalized proteomic analysis. In 2017, Cristobal and coworkers first performed deep proteome profiling of human colon organoids and identified common features shared by the original cancer samples, as well as individual diversity that could aid in personalized cancer treatment (99). Over the past years, the recognition of neoantigens has been an important driver of the clinical activity of T cell-based cancer immunotherapy, and various strategies to identify accurate neoantigens have been pursued (100). In a 2019 study, the authors greatly improved neoantigen identification by using deep learning and large datasets of human leukocyte antigen (HLA) peptide mass spectrometry based on human tumor tissues to create an optimal model of antigen presentation for neoantigen prediction (101). It is likely that tumor organoids could serve as an ideal system to further advance the identification of neoantigens. In 2020, Demmers and coworkers were the first to perform tumor organoid proteomics for the investigation of intrapatient clonal diversity in HLA peptide presentation (102). Single-cell-derived tumor organoids showed high diversity in HLA peptide presentation even within the same cancer patient (102). In summary, organoid-based proteomic analyses are currently feasible and could expand the technical toolbox for precision cancer therapy in the near future.

### Exosomes

Exosomes are small (30-150 nm) extracellular vesicles surrounded by a lipid bilayer membrane and secreted by most eukaryotic cells (103, 104). The components of exosomes, including proteins, nucleic acids (DNA, mRNA, microRNA, lncRNA, etc.), lipids, and metabolites, play important roles in regulating tumor growth, metastasis, metabolism and immune escape (56, 105, 106). Exosomes have been detected in multiple bodily fluids, including blood, urine, cerebrospinal fluid, bile, and saliva, revealing great potential to serve as novel biomarkers for cancer diagnosis (107, 108).

Tumor-derived exosomes are crucial in transferring intercellular signals to modulate the TIME (109, 110). Recently, exosomal PD-L1 has been reported to play vital roles in systemically suppression of the anti-tumor immune response, which illustrates potential mechanism of resistance to PD-L1 blockade (111, 112). Of note, exosomal PD-L1 before and during anti-PD-1 treatment could indicate dynamic states of anti-tumor immunity (111). Therefore, tumor organoid-derived exosomes hold great promise to provide valuable insights regarding immunosurveillance and ultimately cancer immunotherapy. To date, only a few groups have explored the merits of PDTO-derived exosomes in preclinical cancer research. In one study, the authors cocultured esophageal adenocarcinoma-derived exosomes with normal human gastric epithelial organoids (gastroids) and found that exosomal miR-25 and miR-210 could induce an oncogenic phenotype in gastroids (113). Another recent study indicated that PDTO-derived exosomal miRNAs had potential as diagnostic biomarkers for precancerous lesions of colorectal cancer (114). PDTOs could be the source of standardized and scalable production platforms for tumor-derived exosomes, facilitating cancer diagnosis and immunotherapy. However, the availability of organoid-derived exosomes in sufficient quantities, potential contaminants from complex PDTO cultures, and heterogeneous growth rates of PDTOs could be challenging issues, and further research is needed. More studies focusing on the application of organoid-derived exosomes for cancer immunotherapy are underway, and the results are eagerly awaited.

## Conclusion and Future Outlook

The current efficacy of cancer immunotherapy is not satisfactory, and there is a high unmet need for a faithful preclinical model that allows better translation from bench to bedside. Tumor-derived organoids have shown promise for modeling the effects of cancer immunotherapy. While the clinical application of organoid technology is attractive, several significant challenges remain to be overcome. One major bottleneck is that tumor organoids are often derived from biopsies representing only a small part of the entire tumor. In this way, the complexity of the original malignant lesion will always be underrated, and intratumoral heterogeneity could hinder clinical translation. Additionally, the long-term preservation of various immune cells and CAFs needs further optimization.

There are many clinical trials currently ongoing to appraise the merits of PDTOs in precision cancer treatment. Complex tumor organoid culture systems hold promise to unravel the dynamic interactions between the cancer and the immune system and to support drug screening for personalized immunotherapy in the contexts of basic research and clinical trials. As a research tool, tumor organoids currently offer the most accurate *in vitro* system to recapitulate the original human cancer tissues. The applications of genome-wide CRISPR screens, proteomics and exosomes in tumor organoids show great potential for both basic and translational cancer research in the foreseeable future.

## Data Availability Statement

The original contributions presented in the study are included in the article/supplementary material. Further inquiries can be directed to the corresponding authors.

## Author Contributions

JW, CC, and CY conceived this paper. JW, CC, and LW collected the literature. LW and MX drew the schematic diagram. CC and XG prepared the tables. JW, CC, and CY wrote the manuscript. SW, YH, and XM corrected and provide advices for the manuscript and figure. JW, CY, and YS conducted the study supervision and revised the manuscript. All authors read and approved the final manuscript.

## Funding

This work was supported by research grant from the National Natural Science Foundation of China (82103102 to CY). This research was also supported by Zhejiang Provincial Natural Science Foundation of China under Grant No. LQ22H160020 to JW and Grant No. LGF21H150004 to SW.

## Conflict of Interest

The authors declare that the research was conducted in the absence of any commercial or financial relationships that could be construed as a potential conflict of interest.

## Publisher’s Note

All claims expressed in this article are solely those of the authors and do not necessarily represent those of their affiliated organizations, or those of the publisher, the editors and the reviewers. Any product that may be evaluated in this article, or claim that may be made by its manufacturer, is not guaranteed or endorsed by the publisher.
